# Staged Reconstruction for Humeral Osteomyelitis after Severe Crush Injury of the Shoulder: A Case Report

**DOI:** 10.5704/MOJ.2207.020

**Published:** 2022-07

**Authors:** RY Kow, N Mohd-Yusof, MF Abas, CL Low

**Affiliations:** 1 Department of Orthopaedics, Traumatology and Rehabilitation, International Islamic University Malaysia, Kuantan, Malaysia; 2 Department of Plastic Surgery, Hospital Tengku Ampuan Afzan, Kuantan, Malaysia; 3 Department of Radiology, International Islamic University Malaysia, Kuantan, Malaysia

**Keywords:** humerus, osteomyelitis, reconstruction, upper limb, crush injury

## Abstract

The incidence of humeral osteomyelitis is relatively rare as compared to incidence of lower limb osteomyelitis. Despite having no guideline in the management of humeral osteomyelitis, surgeons have utilised their experience in managing lower limb osteomyelitis to treat humeral osteomyelitis. By adhering to principles including thorough debridement of necrotic bone and soft tissue, staged bony and/or soft tissue reconstruction, and targeted antimicrobial therapy, a good outcome can be achieved in the management of humeral osteomyelitis. We report a case of Cierny-Mader type IV proximal humeral osteomyelitis after a severe crush injury of the left shoulder and its subsequent two-stage reconstruction using internal fixation and pedicled Latissimus dorsi musculocutaneous flap.

## Introduction

The incidence of osteomyelitis of the humerus is reported to be 2.6-13.3% of all osteomyelitis cases1,2. It is relatively uncommon as compared to the incidence of lower limb osteomyelitis1. Due to the rarity of humeral osteomyelitis, information on its treatment in the literature is scarce, with most of the studies limited to case series or case reports1. Despite this limitation, the management of humeral osteomyelitis is based on principles derived from our understanding on lower limb osteomyelitis, namely thorough debridement of the necrotic bone and soft tissue, staged bony and/or soft tissue reconstruction and targeted antimicrobial therapy^1,2^.

We report a case of Cierny-Mader type IV proximal humeral osteomyelitis after a severe crush injury of the left shoulder and its subsequent two-stage reconstruction using internal fixation and pedicled Latissimus dorsi musculocutaneous flap.

## Case Report

**Fig. 1:**
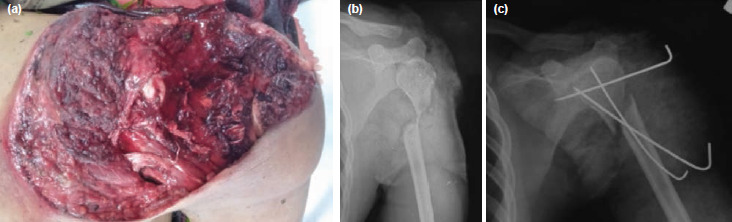
(a) The patient sustained a severe crush injury of the left shoulder. (b) Plain radiograph showed a fracture at the proximal part of the left humerus. (c) After a thorough debridement, the left humerus was temporary held with three 1.6mm Kirschner wires.

MA, an 18-year-old gentleman with no known medical illness, was involved in a motor vehicle accident in which he sustained a severe crush injury of the left shoulder (Fig. 1a and b), closed fracture of proximal third of the left femur and total amputation of left third toe. Emergency wound debridement, arthrotomy washout and Kirschner wiring of the left shoulder (Fig. 1c), refashioning of the left third toe and left tibial pin insertion were performed. Kirschner wire was chosen due to limited space available for Shanz pin placement and severity of the soft tissue conditions. Intra-operatively, the glenohumeral joint capsule was found to be breached and there was total cut of pectoralis minor tendon, long head of biceps tendon, and cocacobrachialis muscle with partial rupture of subscapularis tendon and latissimus dorsi tendon. The axillary nerve was contused but structurally intact. There was no vascular compromise. Five days after the trauma, the patient underwent a second-look debridement. Tissue culture taken intra-operatively grew *Pseudomonas aeruginosa*. Post-operatively, the patient was put on three cycles of negative pressure wound therapy and wound closure with skin graft was performed three weeks after the initial trauma. Interlocking nailing of left femur was performed in the same setting as the left shoulder wound closure. The patient was initially covered with cefuroxime, metronidazole and gentamicin for three days before converting to piperacillin-tazobactam for two weeks based on the tissue microbiology culture and sensitivity (*Pseudomonas aeruginosa*). The Kirschner wires were left in-situ for six weeks prior to removal as there was no sign of infection.

**Fig. 2:**
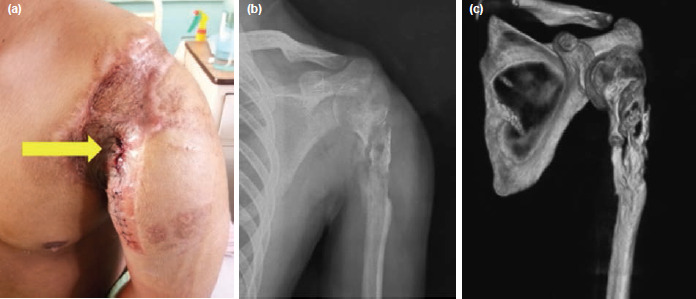
(a) Two months after the initial surgery, he presented with persistent sinus discharge (yellow arrow) at the anterolateral aspect of his left shoulder. (b) Plain radiograph of the left humerus showed sequestrum located at the proximal part of the left humerus. (c) Computed tomography of the left humerus provided a detailed delineation of the sequestrum, thus assisting in the planning of bony resection to remove the sequestrum.

**Fig. 3:**
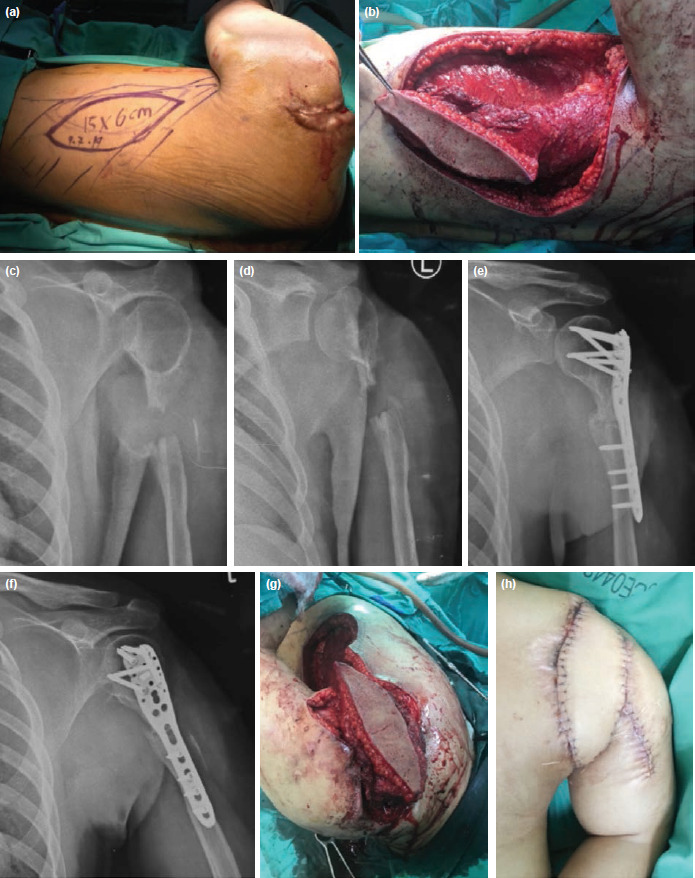

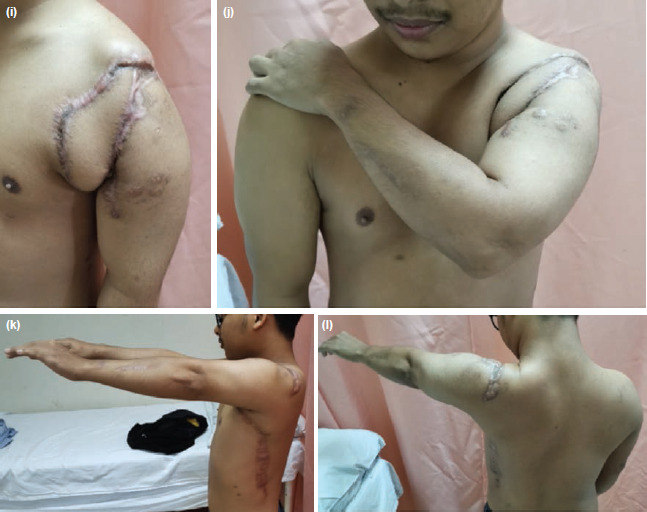
(a) Pre-operative planning of pedicled musculocutaneous latissimus dorsi flap. (b) The pre-planned pedicled musculocutaneous latissimus dorsi flap was raised. (c and d) Plain radiographs (AP and lateral view) showed there was no residual sequestrum after sequestrectomy. (e and f) Final plain radiographs in AP and lateral view. After completion of antibiotics and reducing septic parameters, a second-stage procedure was carried out, involving acute shortening, Proximal Humerus Internal Locking System (PHILOS) insertion, iliac bone grafting and pedicled musculocutaneous latissimus dorsi flap. (g) The pedicled musculocutaneous latissimus dorsi flap was used to cover the soft tissue defect of the shoulder. (h) Post-operatively, the flap appeared to be healthy. (i) Two years after the second stage surgery, the patient achieved both bony union and soft tissue recovery. (j) The shoulder adduction was good. (k) The patient was able to achieve forward flexion up to 90°. (l) The patient was able to achieve abduction up to 100°.

Two months later, he presented with persistent sinus discharge at the anterolateral aspect of his left shoulder (Fig. 2a). Plain radiographs and computed tomography scan revealed a Cierny-Mader type IV osteomyelitis of the left proximal humerus (Fig. 2b and c). He underwent single wound debridement and sequestrectomy as the first-stage procedure. The tissue and bone microbiology culture and sensitivity yielded no growth from the first-stage surgery. After eight weeks of oral antibiotic therapy with cefuroxime and fusidic acid, a second-stage procedure was carried out, involving acute shortening, Proximal Humerus Internal Locking System (PHILOS) insertion, and pedicled musculocutaneous latissimus dorsi flap (Fig. 3a-f). He subsequently achieved both bony union and soft tissue recovery after six months and he was able to return to work. There was no neurology deficit. Two years post trauma, the patient’s Disabilities of the Arm, Shoulder and Hand (DASH) outcome score was 30, indicating mild-to-moderate limitation (Fig. 3g-j). The patient was satisfied with the cosmetic outcome.

## Discussion

In our patient, the risk of infection was very high due to the severity of injury, making upper limb salvage the main priority at that time. The patient sustained severe crush injury with involvement of three out of four main components (bone, soft tissue, and nerve). After a successful limb salvaging surgery, his condition was complicated by diffuse osteomyelitis of the proximal humerus. Diffuse (Cierny-Mader type IV) post-traumatic osteomyelitis of proximal humerus poses a challenge to the treating physician, mainly because the treatment often involves extensive debridement, leaving a large bony and soft tissue defect which requires bone and soft tissue reconstruction^1,2^. Utilising our experience in treating lower limb osteomyelitis, we proceeded with a two-stage surgery. The first-stage procedure focused on elimination of infective foci via adequate debridement, necrotic bone resection and antimicrobial therapy. After debridement and bony resection, the patient was put on U-slab to temporarily stabilise the fracture site. External fixators provide ease in terms of wound management but there is also risk of introducing infection. Furthermore, there was limited spaces to place the Shanz pin proximally. Unlike the tibia and femur bones, relative stability can be achieved with a slab in the upper limb, and the slab can be easily removed for dressing purpose and re-applied after dressing. As the tissue culture yielded no growth of microorganism, patient was covered with oral antibiotics which primarily target gram-positive pathogens such as *Staphylococcus aureus*, a pathogen found in most osteomyelitis cases^1^. A randomised controlled trial (Oral Versus Intravenous Antibiotics for Bone and Joint Infections, OVIVA) shows that oral antibiotic therapy is as effective as parenteral route in the treatment of osteomyelitis^3^. The optimal duration of antibiotics in the treatment of osteomyelitis is still a debated topic but most studies support a duration of six weeks after surgical removal of infective foci^3^. We decided to prescribe the antibiotics up to eight weeks while waiting for the implant for second-stage surgery, during this period we regularly monitored clinical and biochemical septic parameters.

In the second-stage surgery, we had to address two main problems, namely the 2cm bone gap and inadequate soft tissue coverage. For bony reconstruction, we acutely shortened the humerus to reduce the risk of non-union. Limb-length discrepancy at the upper limb is not as debilitating as limb-length discrepancy at the lower limb^1^. There are various reports on good outcomes in bony reconstruction via the Masquelet technique, in which a cement filler is inserted in the first-stage surgery to promote an induced membrane formation. The induced membrane is utilised to contain the bone graft in the second-stage surgery^4^. On top of acting as a mechanical barrier to contain the bone graft, the induced membrane also has osteogenic and osteoinductive properties^3^. Nevertheless, this technique has a steep learning curve and when it is not handled properly, the outcome is guarded^4^. A good soft tissue envelope promotes bony union and prevents the relapse of osteomyelitis. Options around shoulder include pedicled latissimus dorsi flap and scapular flap. We manage to achieve this aim with a pedicled latissimus dorsi flap. Pedicled latissimus dorsi flap is safe and reliable and it provides a large soft tissue coverage for an extensive defect at the shoulder region^5^. As the soft tissue defect is anteriorly located, there will be difficulty in transpositioning the scapular flap to cover the soft tissue defect. On top of that, pedicled latissimus dorsi flap, a myocutaneous flap, offers a better soft tissue cover for the defect as it is well vascularised and the padding it renders is better than scapular flap, a faciocutaneous flap. Having the soft tissue coverage renders the internal fixation feasible. After acute shortening, the Proximal Humerus Internal Locking System (PHILOS) offers absolute stability, making primary healing of the bone possible.

In conclusion, with proper surgical planning and adherence to principles of osteomyelitis management, a patient with diffuse osteomyelitis of the humerus can achieve an excellent clinical outcome.
